# Cardiac arrest after anesthetic management in a patient with hereditary sensory autonomic neuropathy type IV

**DOI:** 10.4103/1658-354X.76486

**Published:** 2011

**Authors:** Yakup Ergül, Bariş Ekici, Sabiha Keskin

**Affiliations:** *Division of Pediatric Neurology of Cerrahpaşa Medical Faculty, Istanbul University, Istanbul, Turkey*

**Keywords:** *Anhydrosis*, *cardiac arrest*, *hereditary sensory autonomic neuropathy type IV*, *hyperprexia*, *self mutilation*

## Abstract

Hereditary sensory autonomic neuropathy type IV is a rare disorder with an autosomal recessive transmission and characterized by self-mutilation due to a lack in pain and heat sensation. Recurrent hyperpyrexia and anhydrosis are seen in patients as a result of a lack of sweat gland innervation. Self-mutilation and insensitivity to pain result in orthopedic complications and patients undergone recurrent surgical interventions with anesthesia. However, these patients are prone to perioperative complications such as hyperthermia, hypothermia, and cardiac complications like bradycardia and hypotension. We report a 5-year-old boy with hereditary sensory autonomic neuropathy type IV, developing hyperpyrexia and cardiac arrest after anesthesia.

## INTRODUCTION

Hereditary sensory autonomic neuropathy type IV (HSAN type IV) which is also known as congenital insensitivity to pain and anhydrosis is characterized by lack of sweating, unexplained fever episodes, and self-mutilation.[1–4] The course of the disease necessitates several surgical interventions to treat soft tissue infections, joint deformities, fractures, digital amputations, and dental complications.[1,5] However, these patients are prone to perioperative complications such as hyperthermia, hypothermia, and cardiac complications like bradycardia and hypotension.[6,7] In this report we present a 5-year-old boy with HSAN type IV who developed anesthetic-induced hyperpyrexia and cardiac arrest.

## CASE REPORT

The patient was a 5-year-old boy who was the third child of a consanguineous marriage of second cousins. The history of pregnancy, labor, and delivery were all unremarkable. His mother was aware that he was not sweating at all and was non-responsive to pain since birth. He was also reported to be hospitalized several times for boots of fever without an underlying cause. The mother recalled that she lost one younger son due to gastroenteritis at age 10 months who also had a dry skin with anhydrosis. The patient had started chewing his limps and fingers following the eruption of his teeth. At age 4, he had been operated for fracture of left caput femoris. Two months after the operation he had to be re-operated for osteomyelitis and abscess formation in the same bone. He developed a cardiac arrest nearly 24 h after the operation and referred to pediatric intensive care unit.

**Figure 1:**
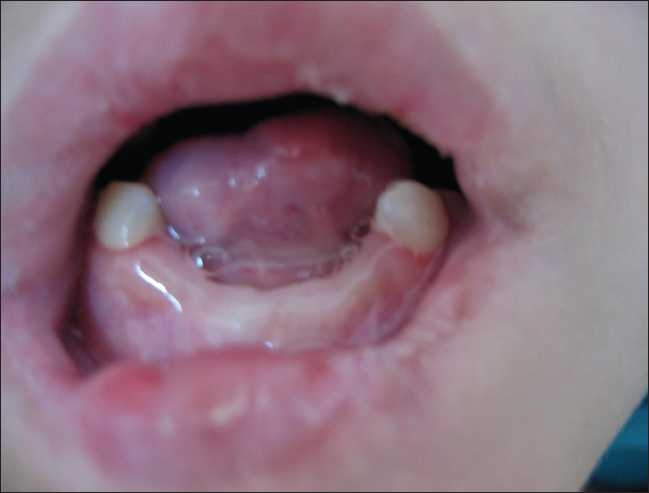
Tissue defect in the tongue, gum, and fingers due to self-mutilation

**Figure 2:**
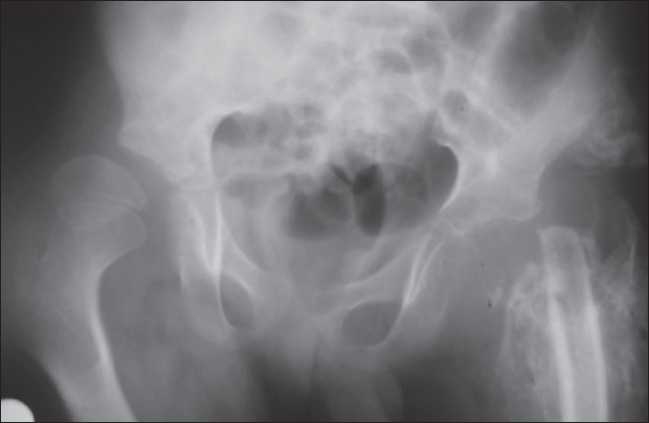
X-ray examination shows necrosis, osteomyelitis, and abscess formation in the left caput femoris

**Figure 3:**
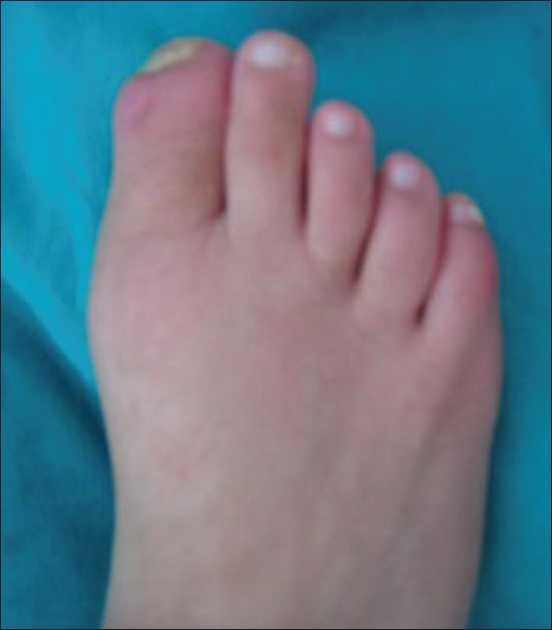
Partial autoamputation in the right toe

On initial evaluation, he was found to have a dry skin and tissue defects in the tongue, gum, and fingers due to self-mutilation [Figure 1]. His X-rays revealed the absence of the left caput femoris due to osteomyelitis and abscess [Figure 2] as well as autoamputations in several fingers and toes [Figure 3]. He was non-responsive to pain with absent pupillary reaction to light. He had several fever attacks (39° C) without any laboratory signs of infection and/or inflammation. He also showed bradycardia which did not respond to atropine treatment.

**Figure 4:**
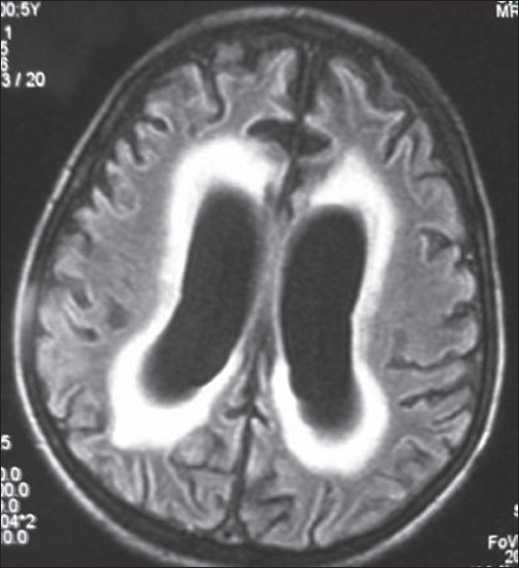
Cranial MRI shows ventricular dilatation and cortical atrophy

He was non-responsive to intradermal histamine and pilocarpine iontophoresis. His whole blood count, blood chemistry, serum and urine amino acids, humoral and cellular immunity tests were all non-diagnostic. His motor and sensory peripheric nerve conduction velocities were also normal. Cranial magnetic resonance imaging showed cortical and supratentorial atrophy with dilatation of the third and fourth ventricules [Figure 4]. He was diagnosed with HSAN type IV based on the clinical and laboratory findings.

## DISCUSSION

Congenital neuropathies are classified into five groups as sensory neural radicular neuropathy, congenital sensorial neuropathy, familial dysautonomy, congenital insensitivity to pain and anhydrosis and congenital pain indifferantion.[2] HSAN IV is extremely rare among most populations with the exception of the Japanese and Israeli-Bedouins.[8] HSAN type IV can be differentiated from other neuropathies by insensitivity to pain and fever, anhydrosis with dry skin, normal sense of touch, diminished or upset deep tendon reflexes, normal corneal reflex response, and lacrimation and finally mental retardation.[5,9] Self-mutilation gives rise to complications like recurrent soft tissue infections, acute or chronic osteomyelitis, and digital autoamputations.[5,9,10] Hyperprexia due to anhydrosis and defects in thermoregulation causes death in 20% of the patients in the first 3 years of life.[1,10] Unresponsiveness of axonal reflexes to intradermal histamine test and lack of sweating by iontophoresis are characteristic findings of HSAN type IV.[5,9] Motor and sensory nerve velocities and responses are usually normal in electromyographic (EMG) examinations.[1,11] Although the light microscopic examination of the peripheric nerves as well as skin biopsy specimens are unrevealing, the electron microscopic examination clearly shows a decrease or absence in the unmyelinized and small myelinized fibriles.[3,5,11] HSAN type IV is a multisystem disorder caused by a mutation in theTrk A gene which results in an abnormal catecholamine metabolism.[12] Molecular studies may not be necessary if the patient presents with the clinical findings but a normal nerve biopsy and EMG.[13]

The presence of anhydrosis, recurrent febrile attacks of unknown origin, self-mutilation, and osteomyelitis with abscess formation in the caput femoris clearly supported the diagnosis in our patient. His unresponsive to the intradermal histamine test was believed to also be confirming the diagnosis. The normal findings from the light microscopic examination of his sural nerve were not exclusive criteria.[5]

Those patients with HSAN type IV frequently need orthopedic and dental operations mostly due to self-mutilation which necessitate the use of anesthetics. Indeed it is also frequently encountered in the patient gastrointestinal and cardiovascular complications such as malaise, emesis, hypotension, bradycardia, and thermoregulation anomalies during perioperative period.[7,14,15] In their study, Rozentsveig *et al,*[7] showed that of the 40 HSAN type IV patients given anesthesia, 20 had moderate hypothermia and 15 had cardiovascular complications. One patient in this study had a cardiac arrest and lived in the vegetative state for 6 days. Okuda *et al*[9] reported postoperative malaise and emesis in 6 and hyperthermia in 4 of the 20 patients with HSAN type IV, none of them showed cardivascular complications. Our case however had been given general anesthesia for two orthopedic operations. Cardiac arrest occurred within 24 h after the second operation on which he was admitted in the intensive care unit. He also developed an attack of bradycardia after cardiac arrest and lasted for 12 h which did not respond to atropine. Atropine-resistant bradycardia was also reported in the literature.[15]

Although our patient showed most of the previously reported clinical findings of HSAN type IV with a history of insensitivity to pain and fever, diagnosis could be done after anesthetically induced severe cardiovascular complication. We believed in the importance of increasing the awareness of the clinical findings and its anesthetically induced complications.
